# Exercise-induced hypoalgesia: A meta-analysis of exercise dosing for the treatment of chronic pain

**DOI:** 10.1371/journal.pone.0210418

**Published:** 2019-01-09

**Authors:** Anna M. Polaski, Amy L. Phelps, Matthew C. Kostek, Kimberly A. Szucs, Benedict J. Kolber

**Affiliations:** 1 Department of Biological Sciences, Duquesne University, Pittsburgh, Pennsylvania, United States of America; 2 Chronic Pain Research Consortium, Duquesne University, Pittsburgh, Pennsylvania, United States of America; 3 Palumbo Donahue School of Business, Duquesne University, Pittsburgh, Pennsylvania, United States of America; 4 Department of Physical Therapy, Duquesne University, Pittsburgh, Pennsylvania, United States of America; 5 Department of Occupational Therapy, Duquesne University, Pittsburgh, Pennsylvania, United States of America; EHESP Paris, FRANCE

## Abstract

**Objective:**

Increasing evidence purports exercise as a first-line therapeutic for the treatment of nearly all forms of chronic pain. However, knowledge of efficacious dosing respective to treatment modality and pain condition is virtually absent in the literature. The purpose of this analysis was to calculate the extent to which exercise treatment shows dose-dependent effects similar to what is seen with pharmacological treatments.

**Methods:**

A recently published comprehensive review of exercise and physical activity for chronic pain in adults was identified in May 2017. This report reviewed different physical activity and exercise interventions and their effectiveness in reducing pain severity and found overall modest effects of exercise in the treatment of pain. We analyzed this existing data set, focusing specifically on the dose of exercise intervention in these studies. We re-analyzed data from 75 studies looking at benefits of time of exercising per week, frequency of exercise per week, duration of intervention (in weeks), and estimated intensity of exercise.

**Results:**

Analysis revealed a significant positive correlation with exercise duration and analgesic effect on neck pain. Multiple linear regression modeling of these data predicted that increasing the frequency of exercise sessions per week is most likely to have a positive effect on chronic pain patients.

**Discussion:**

Modest effects were observed with one significant correlation between duration and pain effect for neck pain. Overall, these results provide insufficient evidence to conclude the presence of a strong dose effect of exercise in pain, but our modeling data provide tes predictions that can be used to design future studies to explicitly test the question of dose in specific patient populations.

## Introduction

Recent estimates claim that chronic pain affects 1.5 billion people worldwide, and these figures are steadily on the rise[1]. In the U.S., chronic pain is thought to affect over 116 million adults[1]; more than diabetes, cancer, and heart disease combined[2]. As a result, chronic pain remains a pervasive medical problem consuming a vast amount of health care resources. In European countries, national healthcare and socioeconomic costs associated with chronic pain total billions of dollars a year[3–5], whereas treatment costs in the U.S. can accrue up to $635 billion dollars annually[2], imposing a substantial economic burden on healthcare systems and society.

Increasing evidence cites exercise as an accessible, cost-effective, and viable therapeutic modality for the treatment of nearly all types of chronic pain conditions[6–17]. Regular physical activity and exercise improve many aspects of a person’s general health, including cardiorespiratory function, mental health, and pain[18, 19]. In general terms, physical activity can include various tasks of daily living, such as work, mobility, leisure, and recreational activities. These are activities that require musculoskeletal activity and energy expenditure. More specifically, exercise is a subset of physical activity, and is defined as structured activity with a goal of improving physical performance and/or health[20]. Common forms of exercise that are studied for the relief of pain include running, walking, resistance training, aquatic exercise, and Tai Chi. Exercise has been found to be effective in relieving pain and benefiting patients’ daily physical function in various chronic musculoskeletal pain disorders, including chronic neck pain[21, 22], osteoarthritis[22, 23], fibromyalgia[24], and chronic low back pain[22, 25]. A dual effect is thus realized for patients with chronic pain because aerobic exercise reduces pain and fatigue as well as improves peak oxygen uptake, health-related quality of life, and physical fitness[26, 27]. Physical activity has been shown to be associated with decreased symptoms of depression and anxiety, further suggesting that exercise could be particularly advantageous in the context of chronic pain comorbid with psychiatric illness[28, 29]. This is particularly important considering that levels of physical activity are inversely correlated with depression symptoms in fibromyalgia patients[30].

Although overall evidence shows exercise to be at least moderately beneficial in chronic pain[31], there exists a number of compliance issues related to the prescription of physical exercise. One of the most pressing issues, especially for individuals with musculoskeletal conditions, is the presence of fear avoidance behavior or kinesiophobia. Additional issues include limited access to training for safe exercise, limited access to equipment, failure to account for the presence of acute pain during exercise, and the lack of data specific for each individual condition. Surprisingly, there is almost a complete lack of studies in the exercise and pain literature testing multiple doses of exercise in a single patient (or control) group.

The four components of dose that can be adjusted for exercise prescription include: 1.) the *frequency* of which exercise is performed in one week, 2.) the *time* in minutes exercises is performed in one week, 3.) the *duration* in weeks that the exercise intervention is performed, and 4.) the intensity of exercise. It is important to note that duration is not typically prescribed or examined as a component of exercise dose, but is included here because evaluating the duration of a study allows for a novel examination of exercise effects from an acute to a chronic perspective. One caveat to this definition of dose is that it does not accurately describe all variables incorporated into resistance exercise programs. Common variables in resistance training interventions include: training volume (i.e. period, frequency, number of sets per exercise, number of repetitions per set), training intensity (i.e. intensity, time under tension) and rest (rest in between sets and repetitions)[32, 33]. However, we chose the 4 variables for dose as mentioned above (frequency, time, duration, intensity), because these factors were common across all forms of exercise assessed in this analysis.

The American College of Sports Medicine recommends 30 minutes of moderate-intensity exercise five days per week (or 150 MET-minutes) in order to maintain cardiorespiratory, musculoskeletal, and neuromotor fitness for healthy adults[34]. Ideally, this dose of exercise would be implemented and maintained long-term for continued health benefits. However, these recommendations may be too high of a starting dose for persons experiencing chronic pain, especially conditions associated with movement-induced pain and movement-associated fear avoidance behavior[35, 36]. It is necessary to critically evaluate the most appropriate dose of exercise for chronic pain.

In this review, we analyze published data evaluating the impact of different exercise modalities across different types of pain. A recent meta-analysis, Geneen et. al [37], was published in the Cochrane Database of Systematic Reviews, which is among the leading resources for meta-analyses in health care. This review assessed physical activity and exercise in chronic pain patients utilizing data from 21 other individual meta- analyses (6495 total studies screened). Rather than starting *de novo*, we took a unique approach of performing additional analyses on this already existing study. This adds consistency to the literature; papers included in the above meta-analysis were already peer-reviewed and had met the Cochrane rigorous criteria for inclusion.

The purpose of the present analysis was to evaluate how changing the measured dose of exercise correlates with a measured pain effect size. In this unique analysis, we treat each study as a single data point in which the measured outcome is the standardized effect size for pain. Data from Geneen et. al[37] was used to conduct correlation analyses with linear and multivariate linear regression evaluating dose of exercise with effect size for pain. The overall objective of this review was to test the hypothesis that the dose of exercise would impact the efficacy of exercise and physical movement-based therapy to reduce chronic pain.

## Materials and methods

A recently published comprehensive review of exercise and physical activity for chronic pain in adults written by Geneen et al [37] was identified in the Cochrane Library in May 2017. This paper reviewed 21 other Cochrane Library Meta-Analyses covering a total of 381 individual studies (of 6495 studies screened) that assessed different physical activity and exercise interventions and their effectiveness in reducing pain severity across eight pain conditions[6, 11–17, 38–51]. All exercise interventions included in this meta-analysis are classified as discrete forms of physical activity by the WHO[20]. The primary literature cited in these Cochrane Meta-Analyses were then organized according to intervention type and chronic pain condition for our analysis.

### Study selection

Studies that were selected for our analysis from Geneen et al[37], met the following inclusion criteria: 1) Randomized control trial (RCT) assessing physical activity or exercise as the intervention, 2) chronic non-cancer pain lasting 3 months or longer[52], 3) patients 18 years or older, 4) meta-analysis must report effect sizes for pain outcome that reflect post-intervention measure for that respective study, and 5) studies were published in peer-reviewed journals. Studies were excluded from analysis if: 1) not published in the English-language, 2) intervention featured multimodal treatments (e.g. walking and weight lifting), 3) study prescribed individualized exercise training plan (e.g. each patient in study received a different plan or dose), or 4) intervention not deemed exercise by WHO definition (i.e. manipulation, mobilization, or passive movement)[20].

### Data extraction

Following selection of studies analyzed in Geneen et al[37], articles were systematically organized based on exercise modality and pain states. Exercise modalities consisted of broader non-mutually exclusive categories such as, strength/resistance training, aerobic exercise (land or water), aquatic exercise, and meditative movement therapy. More specific classification and analysis of exercise programs included walking, jogging, Pilates, tai chi, qigong, motor control exercises, range of motion (ROM) and flexibility exercises, aquatic aerobics, aquatic resistance training, and land aerobic exercise. These exercise interventions are defined as exercise training and not acute physical activity or singular bouts of exercise. Disease states included rheumatoid arthritis (RA), osteoarthritis (OA), fibromyalgia (FMS), low back pain (LBP), intermittent claudication (IC), neck pain (NP), spinal cord injury (SCI), and patellofemoral pain (PFPS).

For each included study, effect sizes, means, standard deviation and 95% confidence interval (C.I.) were extracted from each pain-related outcome measure reported in Geneen et al[37]. To avoid running multiple analyses on the same data sets, we extracted effect sizes that reflected the immediate post-intervention time point; this effect size reflected changes of the exercise group compared to the control. Geneen et al effect sizes were presented as mean differences (e.g. effect size for each group) or standardized mean differences (experimental group versus control group)[53]. In the present analysis, all mean differences were converted to standardized mean differences. Standardized mean differences (Cohen’s d effect sizes) were calculated using the control and experimental group means, standard deviations, and sample sizes reported by referring back to the original primary source[53]. In addition, all standardized effect sizes were converted such that a reduction in pain is presented as a positive effect value.

Pain-related outcome measures included: Visual Analog Scales (VAS)[54], a numerical ratings scale (NRS)[55], McGill Pain Questionnaire (MPQ)[56], Arthritis Impact Measurement Scale 2 (AIMS2)[57], Western Ontario McMaster Osteoarthritis index (WOMAC)[58], the Short Form-36 Health Survey (SF-36) for bodily pain[59], the Health Assessment Questionnaire (HAQ)[60], and the West-Haven Yale Multidimensional Pain Inventory (WHYMPI)[61]. For the surveys, pain effect sizes were calculated from the pain specific section or pain subscale of that survey.

The dosage of the exercise intervention was extracted from each study. The *dose* of exercise was analyzed in three distinct methods. First, dose was defined as being the frequency or bouts at which the exercise was performed within one week (FREQUENCY). Second, dose was defined as the cumulative amount of time (minutes) that the exercise was being completed per week (TIME). Finally, dose was defined as the duration (weeks) that the exercise treatment was implemented in the study (DURATION). For example, if a protocol prescribed an exercise intervention for 3x/week, 30 minutes a session for 4 weeks, the (FREQUENCY) would be 3, (TIME) would be 90 minutes, and (DURATION) would be 4 weeks.

Intensity of exercise was also evaluated in a separate analysis. Intensity was defined as absolute intensity (MET equivalent of the activity) multiplied by the number of minutes performed in one week. MET levels for each activity were derived from The 2011 Compendium of Physical Activities: Tracking Guide[62]. Although a more accurate method for measuring intensity for resistance exercise would incorporate the load, volume, rest periods and order of the exercises, this was not possible due to limited or absent information on these variables in the primary studies.

### Data analysis and statistics

Statistical analyses included a set of univariate analyses followed by multivariate modeling informed by trends observed in the univariate analyses. Univariate correlation analyses were performed to ascertain if there were any significant trends of standardized effect size. This was done by disease states or by exercise modality that might form interactions or dependencies in the independent variables used in the multivariate linear regression modeling to predict significant pain effect. Independent variables in the multivariate modeling included whether the study showed significant results or not, dose measured as TIME, FREQUENCY, and DURATION.

### Univariate analysis

Previously, linear dose-response relationships have been observed with physical activity[63, 64], and linear models have been used to quantify these relationships[64]. Here, we evaluate data by looking at the correlations between the dosage of exercise and the overall analgesic effect (e.g. standardized mean effect size). A Shapiro-Wilk Goodness of Fit for Normality was performed on each of the four pain groups analyzed by the three dose measurements to determine the appropriate statistical test. Linear regressions with Pearson’s Correlation Coefficients were performed using standardized pain effect size and exercise dose for all disease states. p<0.05 was considered statistically significant. Two primary analyses were completed.

Analysis 1 –Pain State—Correlations were compared between studies within the same cohort of pain state classification (e.g. fibromyalgia, low back pain). Here, data were combined across exercise intervention type. Studies with unspecified or insufficient dosage information were not analyzed in regards to that classification of exercise dose. A distinct analysis of exercise dose within some disease states was not completed due to the low number of available studies for certain patient populations.

Analysis 2 –Exercise Type—Correlations were compared between studies within the same cohort of exercise modality (e.g. Pilates, aquatic exercise). Data were combined across pain conditions to determine whether dosing effects of specific exercise types are generalizable across pain conditions.

After evaluating dose by univariate measurements, we also sought to account for effects of exercise intensity. This was done through a “Dose Intensity x Time” analysis. We assessed the relationship between exercise intensity and standardized pain effect size. This was done in a similar fashion to the other univariate analyses above where analyses were performed in respect to pain state and exercise type. Data was evaluated by looking at the correlations between the intensity of exercise and the overall analgesic effect (e.g. standardized mean effect size). Linear regressions with Pearson’s Correlation Coefficients were performed using standardized pain effect size and exercise dose for all disease states. p<0.05 was considered statistically significant.

### Multivariate analysis

In order to control for studies that produced significant effect sizes and to model the effects of the three time-related dose measurements simultaneously, multivariate linear regression modeling was fit using a dummy variable for whether the study showed a significant (p<0.05) pain effect or not plus adding the three main effects of measured dose as TIME, FREQUENCY, and DURATION. Two-way interactions between the three measured dose effects were also added to the model. Selection of the best model fit was determined by significant main effects and interaction effects providing an overall significant model F-statistic (p<0.05) and adjusted R^2^.

## Results

### Characteristics of included studies

**Fig 1:**
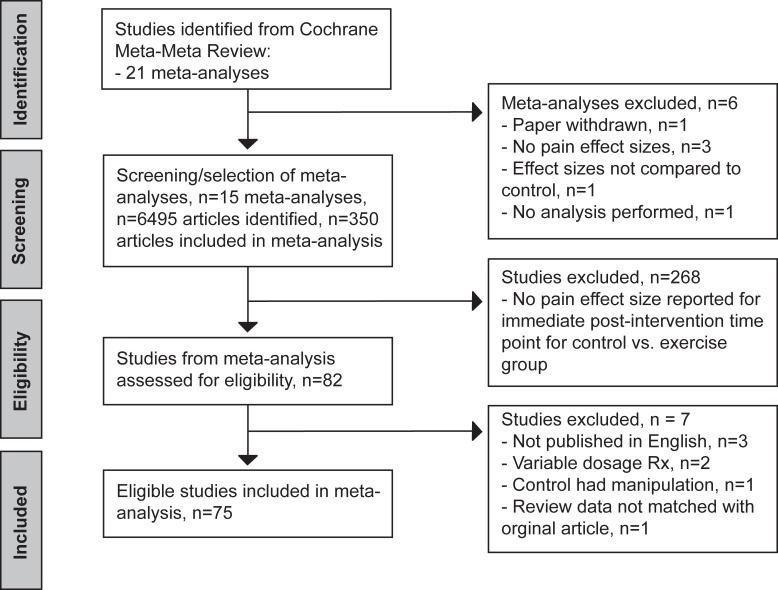
Flow chart diagram showing reference screening and study selection based on exclusion and inclusion criteria.

Of the 381 studies identified in Geneen et al[37], 75 individual studies met the inclusion criteria for our analysis (**Fig 1**). Most of the studies that were omitted from this analysis, but were included in Geneen et al., did not contain an effect size for a pain related outcome, did not report such effect size at the immediate post-intervention time point, and/or did not reflect the relationship for the control vs. exercise group comparison. See **Table 1** for a compilation of each study’s characteristics. Studies identified in more than one Cochrane Review (i.e. a duplicate study) were only analyzed once. Some studies reported a range of TIME (minutes per week) and subsequently IxD (MET-min per week) for the exercise intervention which can be seen in **Table 1**. Due to the variability in dosage, these particular data points were not included in analysis.

**Table 1:** Characteristics and data from studies included in analysis.

INT (broad cat.)	INT (spec. cat.)	METS	Pain State	Review	Authors	Pain outcome	EX (n)	EX Mean (SD)	FREQ. (x/wk)	TIME (min/wk)	IxD (MET-min/wk)	DUR. (wks)	C (n)	C Mean (SD)	Cohen’s d	95% C.I.
Aerobic	Aerobic resistance + ROM /FLEX	4.3	FMS	[6]	[65]	VAS	28	1.5 (1.2)	3x	270–540	1161–2365	6	28	0.5 (1.2)	0.83	[0.28, 1.38]
Aerobic	Bicycle	10.0	OA	[42]	[66]	WOMAC	13	18.6 (13.4)	2x	80–120	800–1200	12	15	34.3 (15.9)	1.03	[0.23, 1.83]
Aerobic	Conditioning exercise	9.0	RA	[11]	[67]	VAS	11	0.2 (0.6)	3x	135–270	1215–2430	104	13	0 (0.5)	-0.35	[-1.16, 0.46]
Aerobic	Ergometer exercise	6.0	FMS	[6]	[68]	VAS	20	2.8 (0.7)	3x	120	720	6	20	1.5 (1.1)	1.34	[0.65, 2.04]
Aerobic	Ergometer exercise	2.8	IC	[13]	[69]	SF-36	32	52.8 (23.5)	2x	80	224	24	32	50.8 (18.5)	0.09	[-0.40, 0.58]
Aerobic	Gymnastics	3.8	NP	[14]	[70]	VAS	22	-9.0 (12.0)	1x	45	171	10	22	-8.0 (18.5)	0.06	[-0.53, 0.65]
Aerobic	Land aerobic	7.3	FMS	[6]	[10]	VAS	51	0.5 (1.4)	7x	70–210	511–1533	16	36	0.5 (1.3)	0.0	[-0.43, 0.43]
Aerobic	Land aerobic	7.3	FMS	[6]	[71]	VAS	20	10.0 (12.7)	3x	135	985.5	14	20	-7.0 (14.6)	1.22	[0.54, 1.90]
Aerobic	Land aerobic	7.3	IC	[13]	[72]	SF-36	17	45.2 (9.8)	2x	120	876	12	12	41.5 (9.8)	0.38	[-0.36, 1.13]
Aerobic	Land or aquatic aerobic	5.5	OA	[42]	[73]	KOOS	26	24.0 (15.0)	3x	180	990	12	26	32.0 (18.0)	0.48	[-0.08, 1.03]
Aerobic	Pole striding	4.8	IC	[13]	[74]	SF-36	11	60.4 (20.5)	3x	90–180	432–864	24	10	49.0 (16.1)	0.61	[-0.26, 1.49]
Aerobic	Qigong	3.0	OA	[42]	[75]	WOMAC	11	71.1 (110.1)	5x	150	450	8	10	138.2 (112.6)	0.58	[-0.30, 1.46]
Aerobic	Qigong	3.0	NP	[14]	[76] (1)	VAS	39	30.8 (16.2)	1-2x	90–180	270–540	24	39	38.9 (18.1)	0.47	[0.02, 0.92]
Aerobic	Qigong	3.0	NP	[14]	[77] (1)	VAS	31	47.4 (30.8)	2x	90	270	12	35	54.9 (28.5)	0.25	[-0.23, 0.73]
Aerobic	Tai Chi	3.0	OA	[41]	[78]	WOMAC	15	5.6 (3.2)	2x	120	360	12	5	9.2 (3.4)	1.08	[0.00, 2.16]
Aerobic	Tai Chi	3.0	OA	[42]	[79]	VAS	22	15.4 (5.7)	3x	120	360	12	19	16.6 (4.7)	0.23	[-0.38, 0.85]
Aerobic	Tai Chi	3.0	OA	[42]	[80]	VAS	79	37.3 (21.1)	n/a	n/a	n/a	6	74	44.4 (23.2)	0.32	[0.0, 0.64]
Aerobic	Walking	4.3	OA	[42]	[81](1)	VAS	144	2.1 (0.6)	3x	180	774	72	75	2.5 (0.6)	0.53	[0.81, 0.24]
Aerobic	Walking	3.5	OA	[42]	[82]	MPQ	17	1.4 (0.9)	7x	n/a	n/a	12	17	1.2 (1.0)	-0.16	[-0.83, 0.52]
Aerobic	Walking	4.3	RA	[11]	[83](1)	AIMS	28	-1.2 (1.9)	3x	180	774	12	28	-0.7 (1.8)	0.27	[-0.26, 0.79]
Aerobic	Walking	3.0	IC	[13]	[84]	SF-36	27	81.5 (18.4)	3x	n/a	n/a	12	26	77.3 (17.8)	0.23	[-0.31, 0.77]
Aerobic	Walking/ jogging	6.0	LBP	[12]	[85]	MPQ	24	22.4 (13.1)	5x	50–100	300–600	8	23	26.8 (13.6)	0.33	[-0.25, 0.91]
Aerobic/ Strength	Bicycle with resistance	11.0	RA	[11]	[86]	VAS	25	0.2 (1.4)	3x	180	1980	12	25	0.9 (1.2)	0.53	[-0.04, 1.09]
Aerobic/ Strength	ROM + FLEX	3.0	PFPS	[17]	[87](1)	VAS	33	0.5 (1.1)	3x	60–180	180–540	16	13	6.6 (1.4)	5.03	[3.82, 6.24]
Aerobic/ Strength	Walking + ROM + FLEX	4.3	OA	[42]	[88]	WOMAC	68	4.9 (3.4)	3x	180	774	8	43	6.2 (4.3)	0.34	[-0.04, 0.73]
Aquatic	Aquatic aerobic	5.5	FMS	[39]	[89]	VAS	17	-18.4 (27.6)	3x	180	990	12	17	1.0 (17.2)	0.84	[0.14, 1.55]
Aquatic	Aquatic aerobic	5.0	FMS	[39]	[90]	VAS	28	-12.0 (23.0)	1x	45	225	20	29	-2.0 (21.0)	0.45	[-0.07, 0.98]
Aquatic	Aquatic aerobic	5.0	FMS	[39]	[91]	VAS	57	-4.9 (12.9)	1x	45	225	20	52	-0.02 (11.9)	0.39	[0.01, 0.76]
Aquatic	Aquatic aerobic	5.3	OA	[38]	[92]	WOMAC	152	-8.5 (3.7)	2x	120	636	52	158	-9.4 (3.5)	0.24	[0.02, 0.47]
Aquatic	Aquatic aerobic	5.5	RA	[11]	[83] (2)	AIMS	40	-0.6 (1.7)	3x	180	990	12	28	-0.7 (1.8)	-0.06	[-0.54, 0.43]
Aquatic	Aquatic aerobic/ resistance	5.0	FMS	[39]	[93]	VAS	15	-3.0 (11.5)	3x	180	900	32	15	2.0 (13.8)	0.39	[-0.33, 1.12]
Aquatic	Aquatic resistance	5.0	OA	[38]	[94]	WOMAC	35	-10.0 (4.0)	3x	n/a	n/a	6	35	10.0 (4.0)	0.0	[-0.47, 0.47]
Aquatic	Aquatic resistance	3.0	OA	[38]	[95]	HAQ	98	-1.4 (0.7)	2x	n/a	n/a	20	117	-1.5 (0.6)	0.12	[-0.15, 0.39]
Aquatic	Aquatic resistance	5.0	OA	[38]	[96]	VAS	21	-43.5 (18.6)	3x	150	750	12	22	-54.9 (25.2)	0.5	[-0.10, 1.11]
Aquatic	Aquatic resistance	5.0	FMS	[39]	[97]	VAS	24	-21.0 (16.5)	3x	105	525	12	22	-18.7 (11.0)	0.16	[-0.42, 0.74]
Aquatic	Aquatic resistance	5.0	FMS	[39]	[98]	VAS	29	-11.4 (11.1)	3x	150–210	750–1050	16	24	3.3 (15.0)	1.14	[0.56, 1.72]
Strength	Aerobic resistance	4.3	OA	[41]	[99]	NRS	45	4.02 (2.9)	1-2x	30–60	129–258	8	43	5.6 (2.8)	0.55	[0.13, 0.98]
Strength	Ergometer exercise	6.0	SCI	[15]	[100]	SF-36	11	4.6 (1.7)	2x	180–240	1080–1440	36	10	6.5 (1.8)	1.09	[0.17, 2.01]
Strength	Motor control exercise	3.5	LBP	[43]	[101]	NRS	77	46.0 (28.0)	1-2x	30–60	105–210	8	77	56.0 (26.0)	0.37	[0.05, 0.69]
Strength	Motor control exercise	3.5	LBP	[43]	[102]	VAS	36	17.2 (15.2)	1x	45	157.5	8	35	31.2 (19.2)	0.81	[0.33, 1.29]
Strength	Motor control exercise	3.5	LBP	[43]	[103]	VAS	21	9.4 (19.3)	5x	100	350	4	21	9.4 (16.2)	0.0	[-0.60, 0.60]
Strength	Pilates	3.0	LBP	[44]	[104]	VAS	8	30.0 (34.0)	2x	120	360	11	9	49.0 (25.0)	0.64	[-0.33, 1.62]
Strength	Pilates	3.0	LBP	[44]	[105]	VAS	20	44.0 (18.0)	3x	120	360	6	14	48.0 (16.0)	0.23	[-0.45, 0.92]
Strength	Pilates	3.0	LBP	[44]	[106]	NRS	43	31.0 (23.0)	2x	120	360	6	43	52.0 (23.0)	0.91	[0.47, 1.36]
Strength	Pilates	3.0	LBP	[44]	[107]	VAS	30	40.4 (24.2)	2x	100	300	8	30	51.6 (25.3)	0.45	[-0.06, 0.96]
Strength	Pilates	3.0	LBP	[44]	[108]	VAS	15	30.9 (16.5)	6x	135	405	8	14	44.6 (15.1)	0.86	[0.10, 1.63]
Strength	Pilates	3.0	LBP	[44]	[109]	NRS	21	18.3 (14.3)	6x	270	810	4	18	33.9 (14.1)	1.10	[0.42, 1.77]
Strength	ROM + FLEX	3.0	SCI	[15]	[110]	VAS	40	1.4 (1.6)	3x	n/a	n/a	12	40	4.2 (2.7)	1.26	[0.78, 1.74]
Strength	ROM + FLEX	2.5	NP	[14]	[76] (2)	VAS	35	28.5 (20.8)	1-2x	n/a	n/a	24	39	38.8 (21.6)	0.49	[0.02, 0.95]
Strength	ROM + FLEX	2.5	NP	[14]	[77] (2)	VAS	35	44.5 (25.7)	2x	90	225	12	35	54.9 (28.5)	0.38	[-0.09, 0.86]
Strength	ROM + FLEX	4.0	PFPS	[17]	[111](1)	VAS	9	2.3 (1.6)	4x	n/a	n/a	8	5	3.5 (1.8)	0.66	[-0.47, 1.79]
Strength	ROM + FLEX	4.0	PFPS	[17]	[111](2)	VAS	9	2.04 (1.7)	4x	n/a	n/a	8	6	3.5 (1.8)	0.78	[-0.31, 1.86]
Strength	ROM + FLEX	2.5	PFPS	[17]	[87](2)	VAS	35	4.0 (1.3)	3x	60	150	16	13	6.6 (1.4)	1.94	[1.20, 2.69]
Strength	Strength	5.0	FMS	[6]	*[112]	VAS	11	24.0 (15.0)	2x	n/a	n/a	17	10	-25.0 (16.4)	3.0	[1.68, 4.32]
Strength	Strength	5.0	FMS	[40]	*[112]	VAS	11	24.0 (15.0)	2x	n/a	n/a	17	10	-25.0 (16.4)	3.0	[1.68, 4.32]
Strength	Strength	3.5	FMS	[40]	[113]	VAS	30	-3.94 (2.0)	3x	180	630	16	30	-2.2 (1.6)	0.99	[0.46, 1.53]
Strength	Strength	3.5	OA	[41]	[114]	VAS	11	37.0 (26.0)	4x	180	630	6	13	47.2 (20.5)	0.43	[-0.39, 1.24]
Strength	Strength	4.0	OA	[41]	[115]	VAS	35	3.6 (2.5)	1x	60	240	8	39	4.1 (2.1)	0.22	[-0.24, 0.67]
Strength	Strength	3.5	OA	[41]	[116]	VAS	35	26.0 (25.9)	1-3x	n/a	n/a	12	33	43.4 (21.6)	0.72	[0.23, 1.21]
Strength	Strength	3.5	OA	[42]	[117]	WOMAC	10	10.8 (4.3)	3x	90	315	6	6	8.3 (4.4)	-0.54	[-1.57, 0.50]
Strength	Strength	3.5	OA	[42]	[118]	VAS	61	22.6 (20.7)	7x	n/a	n/a	8	56	29.6 (23.4)	0.32	[0.05, 0.68]
Strength	Strength	3.5	OA	[42]	[81](2)	VAS	146	2.2 (0.7)	3x	180	630	72	75	2.5 (0.6)	0.36	[0.08, 0.64]
Strength	Strength	5.5	OA	[42]	[119]	WOMAC	20	3.8 (2.7)	3x	n/a	n/a	24	25	4.4 (3.7)	0.18	[-0.41, 0.77]
Strength	Strength	4.5	OA	[42]	[120]	WOMAC	68	4.8 (3.1)	3x	90–150	405–675	8	30	7.1 (3.4)	0.71	[0.27, 1.16]
Strength	Strength	3.5	OA	[42]	[121]	WOMAC	36	4.2 (3.0)	3x	150	525	8	36	7.3 (3.4)	0.96	[0.47, 1.45]
Strength	Strength	3.0	NP	[14]	[122]	VAS	10	3.0 (2.8)	7x	n/a	n/a	2	10	2.6 (2.9)	-0.13	[-1.01, 0.74]
Strength	Strength	2.5	NP	[14]	[123]	VAS	28	1.9 (1.8)	7x	n/a	n/a	6	33	1.7 (1.7)	-0.11	[-0.62, 0.39]
Strength	Strength	5.0	NP	[14]	[124]	VAS	135	2.9 (2.6)	3x	90	450	12	130	2.7 (2.5)	-0.08	[-0.32, 0.16]
Strength	Strength	5.0	LBP	[12]	[125]	WHYMPI	31	29.0 (17.0)	1-2x	n/a	n/a	10	23	41.0 (15.0)	0.74	[0.18, 1.30]
Strength	Strength	3.0	LBP	[12]	[126]	VAS	34	23.0 (16.0)	1-2x	60–120	180–360	13	35	24.0 (17.0)	0.06	[-0.41, 0.53]
Strength	Strength	3.0	LBP	[12]	[127]	MPQ	10	26.0 (17.0)	2x	n/a	n/a	4	10	30.0 (16.4)	0.24	[-0.64, 1.12]
Strength	Strength + ROM + FLEX	3.5	OA	[41]	[128]	WOMAC	55	20.6 (17.2)	2-3x	n/a	n/a	12	54	25.3 (18.5)	0.26	[-0.12, 0.64]
Strength	Strength + ROM + FLEX	5.0	OA	[41]	[129]	WOMAC	60	24.1 (21.7)	1x	45	225	12	58	27.8 (19.8)	0.18	[-0.18, 0.54]
Strength	Strength + ROM + FLEX	3.5	OA	[42]	[130]	WOMAC	25	7.0 (7.5)	1x	45	157.5	4	25	10.0 (7.5)	0.39	[-0.17, 0.95]
Strength	Strength + ROM + FLEX	4.0	OA	[42]	[131]	VAS	25	38.0 (12.5)	2x	100	400	8	27	39.7 (12.0)	0.14	[-0.41, 0.68]
Strength	Strength + ROM + FLEX	3.5	LBP	[12]	[132]	VAS	14	6.0 (17.2)	2x	90	315	16	19	37.0 (17.2)	1.80	[0.99, 2.62]

INT shows categories for intervention type.

(#) denotes the intervention group for studies that included multiple interventions.

* denotes duplicates found.

EX, exercise (group); FREQ, # of exercise sessions per week; TIME, minutes of exercise per week; IxD, intensity x dose MET-minutes of intervention per week; DUR, # of weeks the intervention lasts; C, control (group); SD, standard deviation; ROM, range of motion exercise; FLEX, flexibility exercise; RA, rheumatoid arthritis; PFPS, patellofemoral pain syndrome; OA, osteoarthritis; FMS, fibromyalgia syndrome; IC, intermittent claudication; NP, neck pain; LBP, low back pain; SCI, spinal cord injury; VAS, visual analog scale; WOMAC, Western Ontario McMaster Osteoarthritis index; SF-36, The Short Form (36) Health Survey; AIMS, Arthritis Impact Measurement Scale; MPQ, McGill Pain Questionnaire; KOOS, Knee Injury and Osteoarthritis Outcome Score; HAQ, The Health Assessment Questionnaire; NRS, Numerical Ratings Scale; WHYMPI, West Haven-Yale Multidimensional Pain Inventory. Cohen’s d is represented as standardized mean effect sizes.

Most of the studies included in this review demonstrated some positive benefits of exercise on pain outcomes (69 of 75 studies); of which 30 were statistically significant. Of the statistically non-significant studies, 39 of 45 described positive trending benefits of exercise while only six studies reported worse pain with exercise[67, 82, 83, 117, 122, 123].

### Risk of bias analysis

**Fig 2:**
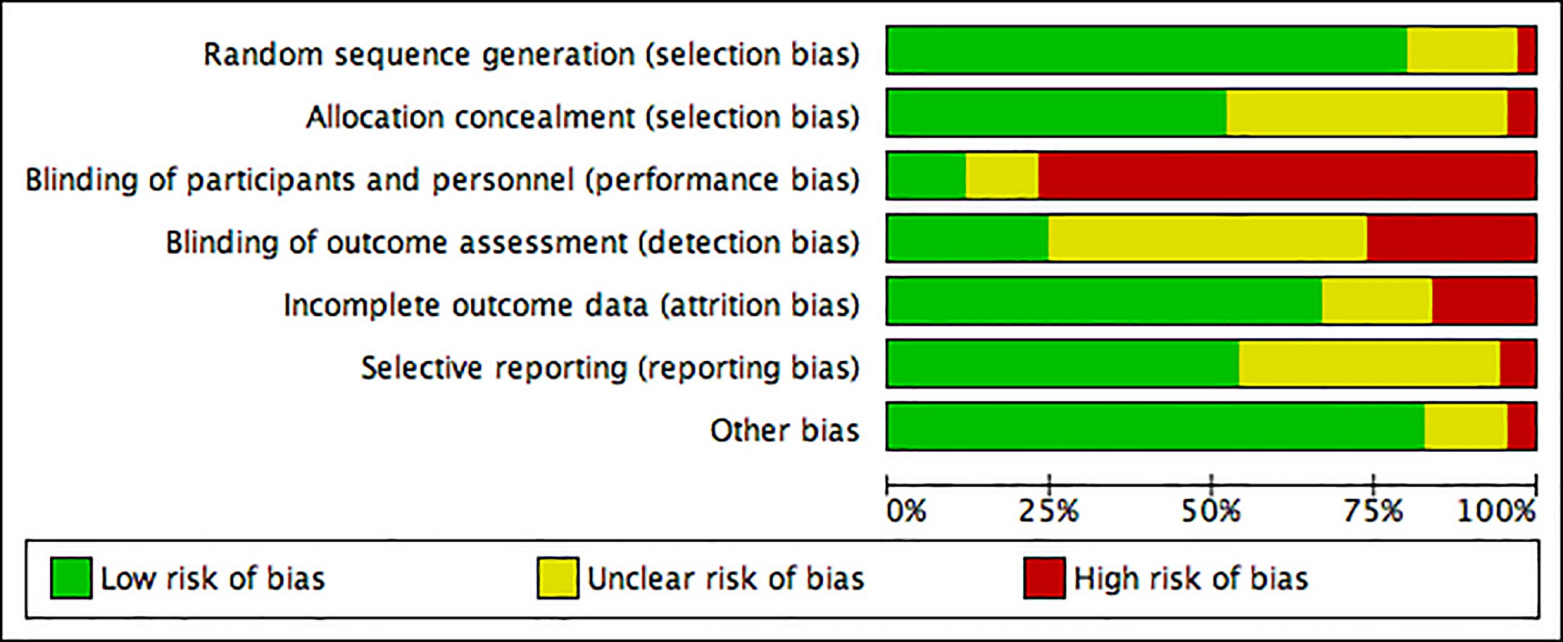
‘Risk of bias’ graph: Review authors’ assessments for each risk of bias item presented as percentages across all included studies.

We assessed the risk of bias of the 75 included studies in accordance with The Cochrane Collaboration’s recommended methods[133] using the Review Manager assessment tool[134]. We evaluated articles according to the following domains: 1) random sequence generation, 2) allocation concealment, 3) blinding of participants and personnel, 4) blinding of outcome assessment, 5) incomplete outcome data, 6) selective outcome reporting and 7) other biases (i.e. baseline imbalances between allocation groups in participant characteristics. **Fig 2** was generated using Cochrane’s ‘risk of bias’ tool to provide summary assessments of the risk of bias. Random sequence generation was adequately described in over 75% of included studies. Low risk of bias for allocation concealment, incomplete outcome data, selective reporting and other bias existed in over 50% of the studies. A high risk of bias was found in over 75% of the studies due to lack of blinding of participants and personnel (performance bias), which was unavoidable in most studies due to the nature of the interventions. Also present in 25% of the included studies were higher levels of detection bias due to participants being un-blinded for self-assessment outcomes (i.e. VAS pain ratings, and other surveys).

### Analysis by pain state

We began by first analyzing the impact of dose for each specific chronic pain disease state combining data across different types of exercise for a single state. Pain states for these analyses included NP[70, 76, 77, 122–124], FMS[10, 65, 68, 71, 89–91, 93, 97, 98, 112, 113], OA[66, 73, 75, 78–82, 88, 92, 94–96, 99, 114–121, 128–131], and LBP[85, 101–109, 125, 126, 132]. Analyses for the remaining pain states; RA, PFPS, IC and SCI, were not performed due to having too few studies. Shapiro-Wilk Goodness of Fit for Normality revealed all pain state analyses to meet the normality assumption, with the exception of the analysis for FMS with FREQUENCY and DURATION. Pearson’s correlations were performed for the three aspects of exercise dose (i.e. FREQUENCY, TIME, and DURATION) in relation to pain effect sizes for each respective study.

**Fig 3:**
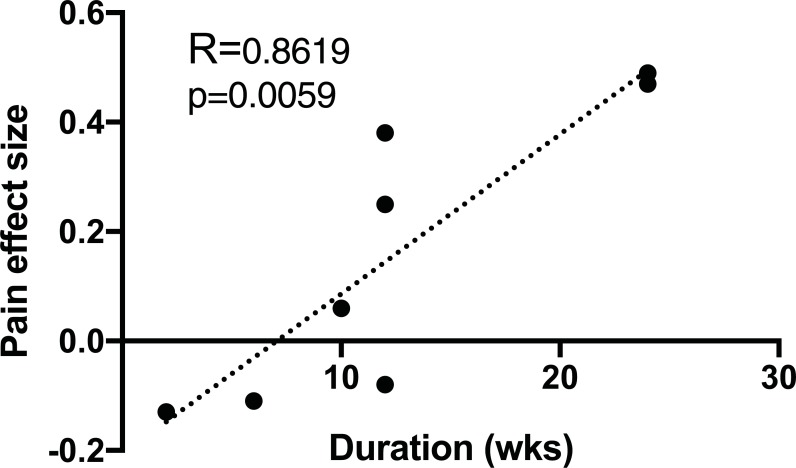
Pain effect size vs. DURATION of exercise for neck pain patients for all exercise modalities. DURATION of intervention is measured in weeks, n = 8, shows a statistically significant positive correlation with analgesic effect (p = 0.0059). R represents Pearson’s correlation coefficient. Dotted line represents line of best fit.

Data for neck pain patients showed a statistically significant positive correlation for DURATION (R = 0.8619, p = 0.0059, n = 8) of intervention with analgesic effect (**Fig 3**). Longer duration studies were associated with more positive analgesic effects. See **Table 2** for a comprehensive list of all analyses performed by pain state.

**Table 2:** Comprehensive list of results for all univariate correlation analyses performed for pain effect size versus dose of exercise intervention.

	R (N)		
Analyses	FREQUENCY	TIME	DURATION
Neck pain (NP)	-0.6969 (6)	0.3035 (4)	**0.8619 (8)
Fibromyalgia (FMS)	-0.2894 (12)	0.3739 (8)	-0.1541 (13)
Osteoarthritis (OA)	-0.1540 (23)	0.3703 (16)	0.0165 (27)
Low back pain (LBP)	-0.1979 (10)	0.1519 (9)	0.3819 (13)
Aquatic exercise	0.0948 (12)	0.3519 (10)	-0.0458 (12)
Aerobic exercise	-0.0585 (35)	0.2633 (26)	-0.1002 (36)
Strength training	-0.1254 (41)	0.1202 (27)	0.1181 (49)
Meditative movement	0.3453 (10)	0.5610 (10)	-0.2183 (12)
Pilates-only	0.5624 (6)	0.6718 (6)	-0.3286 (6)
Walking/jogging	-0.0074 (8)	-0.0725 (7)	-0.0392 (9)
Aquatic aerobic	0.0194 (7)	0.0088 (7)	-0.2308 (7)

Analysis grouping is shown in the left column. R represents value of Pearson’s correlation coefficient. (N) represents the number of individual studies included in the analysis.

**p<0.01.

### Analysis by exercise type

Next, we analyzed the impact of dose on pain effect by evaluating each exercise type combined across different pain conditions for a single exercise type. Initial analyses were performed by grouping exercise modalities into broader categories (i.e. aquatic[73, 83, 89–98], aerobic[10, 65–93, 73–93, 99, 104–109, 135], and strengthening exercise[65, 76, 77, 81, 85, 87, 88, 93–132]).

A secondary analysis was performed by grouping exercise interventions into more specific categories, with the aim of reducing experimental variability between exercise interventions. These categories consisted of meditative movement-based therapies[75–80, 104–109] (i.e. Pilates, tai chi and qigong), Pilates-only[104–109], walking/jogging[67, 74, 81–85, 88, 135], and aquatic aerobics[73, 83, 89–93]. Although there were other specific therapy types present in this cohort of studies, they were of an insufficient sample size to perform separate analyses. We found no statistically significant correlations for the primary or secondary analysis. See **Table 2** for the complete list of results for analyses done by exercise type.

### Multivariate analysis of dose interactions

Following the marginal effect seen in the univariate analysis, we sought to account for potential connections between the dose variables. We reasoned that FREQUENCY, TIME, and DURATION may interact to influence pain effect size. In developing this model, we also considered whether a study actually showed a statistically significant effect. Overall, the multivariate analysis allows us to predict the impact of varying one dose variable on the pain effect.

**Table 3** shows the results of the multivariate linear regression modeling. We found a statistically significant main effect of whether the study showed a significant effect size or not, a statistically significant main effect of dose as measured by FREQUENCY and by TIME as well as, statistically significant interaction effects of TIME in minutes per week by DURATION, and FREQUENCY per week by DURATION. The linear regression equation is given by:

StudyPainEffect=0.374−[0.36FREQ]+[0.01TIME]−[0.03DUR]+[0.743Sig]−[0.001Dur*Time]+[0.04Dur*Freq]

It is noted that due to missing data, mostly in capturing TIME in minutes per week, that only 43 of 75 studies were analyzed to produce this equation. The ratio of non-significant studies to significant studies included in these 43 reflected a similar 2–1 ratio of the total 75 studies selected. Of note, studies with both positive and negative effect sizes were used to develop this model.

**Table 3:** Results of multivariate linear regression modeling for dose interactions.

Parameter	Estimate	Std. Err.	DF	T-Stat	p-value
Intercept	0.374	0.244	36	1.536	0.1333
Dose: FREQUENCY (x/wk)	-0.357	0.122	36	-2.913	0.0061
Dose: TIME (min/wk)	0.007	0.003	36	2.660	0.0116
Dose: DURATION (wks)	-0.026	0.018	36	-1.443	0.1575
Sig	0.743	0.114	36	6.516	<0.0001
Dur*Time	-0.001	0.0002	36	-2.766	0.0089
Dur*Freq	0.042	0.014	36	2.970	0.0053

Shown are the multiple linear regression coefficients. Model F-statistic = 9.628, p<0.001. Adjusted R^2^ = 0.552.

The adjusted R^2^ value of this analysis is 0.552 meaning that 55.2% of the variation in standardized effect size is due to the factors represented in this model. Much of that is naturally attributed to studies which resulted in a significant effect size; however, the model does indicate that even for those studies which did not result in a significant effect size, changing exercise dose does significantly influence measured pain outcomes.

The model predicts that for individual studies which resulted in a significant pain effect, one would expect to see an increase of 0.743 standardized effect size as indicated by the coefficient for the variable “Sig.” However, the statistically significant coefficients for the interaction of TIME by DURATION and FREQUENCY by DURATION (not the main effects coefficients) are the driving numerical forces in this model when using it to predict changes in analgesic effect between different exercise dosing. For all studies that resulted in a significant pain effect and that applied a similar exercise dose as measured in FREQUENCY per week and DURATION, increasing the TIME measured in minutes per week results in a statistically significant increased standardized effect size; this predicted increase is countered by the negative interaction effect of DURATION by TIME. Thus, the overall net effect of increasing TIME in minutes per week predicts a decreased analgesic effect.

For all studies that resulted in a significant pain effect and applied a similar exercise dose as measured by TIME in minutes per week and DURATION, increasing the FREQUENCY per week results in a statistically significant decreased effect size, however this predicted decrease is countered by the positive interaction effect of the exercise regimen duration by frequency. Thus, increasing FREQUENCY of the exercise performed per week predicts an overall net increased analgesic effect. Because of this, it is crucial to take all variables into account when thinking about frequency. Similar effects of changing doses are predicted when using this model for those studies in this meta-analysis which reported non-significant pain effect sizes.

**Table 4** gives an example of how the standardized effect size is predicted to change between studies that produced a significant result and those that did not produce a significant result when changing dose as measured by TIME, FREQUENCY and DURATION. In the 43 studies used to develop the model above, the average TIME exercised was about 120 minutes per week, the average FREQUENCY was 3 times per week and the average DURATION was about 15 weeks. Given these values, the multivariate model predicts a pain effect of 0.8 for studies that resulted in a significant pain effect compared to 0.04 for studies that did not result in a significant pain effect. An effect greater than 0 would be indicative of a positive analgesic effect.

**Table 4:** Predicted pain effect values using the multiple linear regression model for significant and non-significant studies.

		Intercept	FREQUENCY (x/wk)	TIME (min/wk)	DURATION (wks)	Dur*Time	Dur*Freq	Pain Effect Sig	Pain Effect Not Sig
	Coefficient	0.374	-0.36	0.01	-0.03	-0.001	0.04	0.743	0
**Change** **FREQ** **by 1 day**									
	Less 2 day		**1**	120	15	1800	15	0.307	-0.436
	Less 1 day		**2**	120	15	1800	30	0.547	-0.196
	Average Values		**3**	120	15	1800	45	0.787	0.044
	Add 1 day		**4**	120	15	1800	60	1.027	0.284
	Add 2 day		**5**	120	15	1800	75	1.267	0.524
	Add 3 day		**6**	120	15	1800	90	1.507	0.764
**Change** **TIME** **by 30 min**									
	Less 1.5 hr		3	**30**	15	450	45	1.237	0.494
	Less 1 hr		3	**60**	15	900	45	1.087	0.344
	Less 0.5 hr		3	**90**	15	1350	45	0.937	0.194
	Average Values		3	**120**	15	1800	45	0.787	0.044
	Add 0.5 hr		3	**150**	15	2250	45	0.637	-0.106
	Add 1 hr		3	**180**	15	2700	45	0.487	-0.256
	Add 1.5 hr		3	**210**	15	3150	45	0.337	-0.406
**Change** **DURATION** **by 1 wk**									
	Less 6 wk		3	120	**9**	1080	27	0.967	0.224
	Less 4 wk		3	120	**11**	1320	33	0.907	0.164
	Less 2 wk		3	120	**13**	1560	39	0.847	0.104
	Average Values		3	120	**15**	1800	45	0.787	0.044
	Add 2 wk		3	120	**17**	2040	51	0.727	-0.016
	Add 4 wk		3	120	**19**	2280	57	0.667	-0.076
	Add 6 wk		3	120	**21**	2520	63	0.607	-0.136

The last two columns show predicted pain effect sizes for significant studies versus non-significant studies as a result of the exercise prescription shown in the second column. The highlighted cells indicate the variables being changed compared to the average values from the included studies.

Using the model, one can predict the impact of varying one of the three dose variables while keeping the other variables consistent with the average values (**Table 4**). In this way, one can evaluate the relative impact of varying FREQUENCY, TIME or DURATION on predicted pain effect in a hypothetical study. For example, increasing FREQUENCY per week to 6 times per week while holding TIME and DURATION constant results in a predicted pain effect of 1.5 for significant studies compared to the average of 0.8 effect, and 0.8 for non-significant studies compared to the average of 0.04. In other words, doubling the number of exercise bouts from 3 to 6 times per week suggests that the predicted pain effect would increase even in studies that did not result in a significant difference between the control and treatment groups.

In contrast, increasing TIME exercised from the group average of 120 minutes to 210 minutes per week, while holding FREQUENCY and DURATION constant resulted in smaller and slightly negative predicted pain effects of 0.3 and -0.4 for significant and non-significant studies, respectively. Decreasing the TIME spent exercising to 30 minutes per week predicted positive pain effects of 1.2 for significant studies and 0.5 for non-significant studies. Similar predictions were observed while manipulating DURATION (**Table 4**).

### Univariate analysis of dose intensity

Thus far, our analysis has shown a single univariate correlation with exercise study DURATION and neck pain effect. We also found some potentially interesting predictions from a multivariate analysis describing the interactions between different aspects of exercise dose. To complement this simplified analysis of dose and pain effect, we also sought to account for the intensity of exercise in an additional univariate analysis. Here, the intensity of each exercise (MET) was estimated and combined with dose of exercise (in TIME). This combined value (INTENSITYxTIME) was used to evaluate the intensity of exercise because the basic estimated MET intensity (from The 2011 Compendium of Physical Activities: Tracking Guide[62]) for some types of exercise (e.g. qigong, tai chi and Pilates) were identical for all studies even though those studies varied considerably on other aspects of dose. After determining the INTENSITYxTIME of exercise for each study that this could be calculated for (n = 43), we performed a new set of univariate analyses calculating the correlation between effect size and INTENSITYxTIME for studies grouped either by pain state or exercise type as described above. We found no statistically significant relationships in this analysis (**Table 5**).

**Table 5:** Results of univariate correlation analyses performed for pain effect size versus INTENSITY x TIME.

	R (N)
Analyses	INTENSITY x DOSE
Neck pain (NP)	-0.4768 (5)
Fibromyalgia (FMS)	0.5178 (8)
Osteoarthritis (OA)	0.1426 (16)
Low back pain (LBP)	0.1727 (9)
Aquatic exercise	0.0631 (11)
Aerobic exercise	0.1482 (28)
Strength training	-0.0559 (33)
Meditative movement	0.5610 (10)
Pilates-only	0.6718 (6)
Walking/jogging	0.0001 (4)
Aquatic aerobic	0.0126 (7)

Analysis grouping is shown in the left column. Dose of exercise is represented by TIME in minutes per week. R represents value of Pearson’s correlation coefficient. N represents the number of individual studies included in the analysis.

## Discussion

Prescribing exercise as a first-line therapy for chronic pain presents numerous challenges. One significant challenge is determining the exercise therapy best suited for the chronic pain condition and subsequently for each individual patient. It is known that low-to moderate-intensity exercise (50–60% of maximum heart rate) is sufficient to improve chronic pain symptoms[31]. Although there is a significant amount of evidence in the literature suggesting exercise as an efficacious modality for the treatment of chronic pain, there is virtually no knowledge of the appropriate dose of exercise for a given disease or patient type. That is, almost all studies compare a single dose of exercise to control conditions or other alternative treatments. This is a notable and substantive gap in the use of exercise as an evidence-based therapy. The lack of dosing studies for exercise means that patients may not be receiving the optimal therapy and/or be receiving a therapy that actually increases pain. As described above, we have correlated the pain or analgesic effect size seen in patients with the prescribed dose to address this clinically-relevant question.

Although the linear regression analysis of this data found only one significant correlation; Neck pain effect vs. DURATION, the multivariate linear regression modeling allowed us to weigh the relative impact of FREQUENCY vs. TIME vs. DURATION on positive and negative pain effects (i.e. patients getting better or worse). When comparing changes to single dose measurements (FREQUENCY, TIME, or DURATION) while keeping the other variables constant, we found a substantive increased analgesic effect when frequency per week was increased. Conversely, when TIME in minutes exercised per week or DURATION of study was increased, the model predicts a decreased analgesic effect. The significant interactions of TIME and FREQUENCY with DURATION indicate that more specific designed studies are needed to capture optimal treatment exercise plans and that these are likely to vary between disease states and exercise modalities. Generally, the model does support a modicum that daily exercise is likely to support an analgesic effect.

### Dose effects in exercise

The general idea of dose effects in exercise is not controversial. Experimentally, several groups have found dose effects in acute pain, as well as effects of exercise dose in non-pain health-related contexts. Three notable studies have discovered a dose response of isometric contraction on acute pain perception in healthy human participants[136–138]. Hoffman et al. has shown dose effects for intensity and length of exercise session for aerobic exercise-induced analgesia[139]. However, unlike other studies evaluated in the present review that evaluate exercise training, these studies assessed single bouts of physical activity or acute exercise effects on pain. In chronic pain populations, some reccommendations have been provided in regards to exercise dose. In patients with knee OA, aerobic exercise programs were found to have analgesic effects dependent on frequency of sessions, with more sessions having a positive impact on pain reductions.[140]. Macfarlane et al. describes typical protocols used in fibromyalgia research as interventions performed for 20 minutes or greater once a day or 10 minutes or more twice a day, 2–3 days per week[141]. Others have also shown exercise dose-like effects on pain tolerance and pain threshold when comparing triathletes to amateur exercisers[142], and regular runners to normally active controls, respectively[143]. Other groups have found exercise to have positive dose-related effects on cardiovascular health[144], metabolism[144], maximum strength[145, 146] body composition[147], cognition[148, 149], mental health[150], depression[151] and estrogen levels[152]. Similar to the present analysis, these studies evaluated exercise dose in the context of frequency of exercise bouts per week and time (minutes) per week. Likewise, these studies assessed treadmill exercise, cycling, aerobic exercise and strength training, which were comparable to the modalities utilized in the studies that we investigated. Overall, the evidence of dose effects in multiple contexts of exercise suggests that other explanations may be responsible for the lack of large effects in our analyses. In a general context, there is a growing body of literature to support positive effects of exercise on numerous physiological and psychological outcomes[19].

Several animal studies have suggested dose effects of exercise in the context of metabolism and cardiovascular health[144] as mentioned above, as well as in the context of pain[153–156]. In a model of neuropathic pain, forced treadmill running was found to reverse tactile hypersensitivity in an intensity-dependent manner, where low intensity speed (10 m/min) corresponded to walking and high intensity speed (16 m/min) corresponded with running[153]. Rats with free access to running wheels were found to have increased pain thrsholds that were positively correlated to the amount of running activity that was performed[156]. In a model of non-inflammatory muscle pain, five days of physical activity had no effect on pain, while eight weeks of the intervention prevented primary and secondary hyperalgesia, indicating that chronic exercise had positive effects on pain[154]. Comparing across studies, while two weeks of voluntary wheel running failed to reverse hyperalgesia in models of acute inflammatory pain and neuropathic pain[157], six weeks of voluntary wheel running prevented and reversed hyperalgesia in a neuropathic pain model[155]. Assessment of the animal exercise literature is crucial, since it allows direct comparison of the effects of exercise dosing within and across studies in a similarly controlled environment.

If dose effects of exercise do exist, the question remains why we were unable to see substantive evidence of these effects in our linear regression analysis. One potential explanation for the lack of effect could be due to similarity in the doses across studies. However, ranges were quite variable for each aspect of exercise dose; FREQUENCY: 1–7 bouts per week; TIME: 45–540 minutes per week; DURATION: 4–104 weeks of intervention. This would suggest that dose similarity is not a reason for null effects. For the first univariate analysis, studies were grouped by exercise therapy across pain states and for the second analysis, studies were classified by pain state across exercise therapy types. This can greatly contribute to the variability amongst studies in their respective data sets. One way to account for this is to perform a sub-analysis by exercise type and pain state (i.e. Pilates exercise for low back pain patients). However, in doing so, the number of studies in each analysis greatly decreases, which reduces confidence in any significant changes observed. In addition, the studies included in this analysis were carried out at different institutions using multiple enrollment and monitoring methods which also contributes to the variability between studies. Other potential explanations for paucity of positive linear regression effects could be that, 1) these studies are incorporating a dose of exercise above or at a low threshold for exercise benefit[158, 159], 2) the specific mode of therapy may be an ineffective treatment method respective to patient pain condition, 3) exercise of any type may be generally beneficial and dose may have minimal effects in pain patients, and/or 4) the effects are patient specific.

The final explanation of why so few linear regression effects were found here is that it is possible that exercise does not actually have a dose effect in the context of pain. In comparing high intensity versus low intensity exercise in OA patients, one group’s analysis found a lack of short-term improvements in pain and physical function with higher intensity exercise[50]. However, the authors acknowledge this as low quality evidence due to risk of bias and small number of studies and recommend additional studies investigating the dose-response relationship[50].

Although the multivariate modeling suggests dose effects, some animal dosing studies suggest a null effect of exercise dose in pain. In the context of neuropathic pain, Stagg et al. reported significant dose effects in respect to treadmill exercise intensity (low vs high), but not in frequency (3 vs. 5 days/week)[153]. While eight weeks of voluntary wheel running showed analgesic effects in models of chronic muscle pain and exercise-induced pain, these effects were absent in the context of acute inflammatory muscle pain[154]. Six weeks of voluntary wheel running was found to prevent allodynia in a model of neuropathic pain; the distance traveled by the animal running on the wheel did not correlate with allodynia reduction[155]. In uninjured animals, one, two, or four weeks of voluntary wheel running did not alter acute nociceptive thresholds as measured by thermal and mechanical sensitivity assays[157]. In addition, free-access to a running wheel for 2 hours/night for either one, two, or four weeks or 12 hours/night for two weeks did not prevent nocifensive responses to acute formalin-induced inflammatory pain-like behavior and failed to improve mechanical hypersensitivity in a model of neuropathic pain[157]. Although some studies show null or absent effects of exercise dose on pain, these data may be affected by the nature of the pain injury, the intensity or duration of the exercise, or the timing of the intervention in respect to injury.

### Strengths and limitations

All studies included in this analysis were derived from reviews published in *The Cochrane Database of Systematic Reviews* of the Cochrane Library. Because of this, all reviews have been prepared in accordance with the strict guidelines detailed in the *Cochrane Handbook for Systematic Reviews of Interventions*[160]. In addition, the methodological quality of each review was evaluated by criteria specified in the assessment of multiple systematic reviews (AMSTAR) measurement tool[161]. All 21 reviews included in Geneen et al. scored well with the AMSTAR assessment[37]. All exercise interventions considered in this meta-analysis are defined forms of physical activity by the WHO[20], many of which are highly accessible to the general public and do not require extensive equipment or training.

While this analysis provides valuable data for exercise dosing in chronic pain, the review has drawbacks. As previously mentioned, studies included in this analysis were performed in different settings using various enrollment and examination techniques. Sample sizes for some study groups were low. High risk of performance bias (i.e. subjects were not blinded to whether they were receiving the treatment) was also present in the majority of these studies, which may have an impact on the lack of significant findings. Additionally, dosing for resistance exercise did not include all necessary variables, as previously mentioned in the introduction. This is a major caveat to the results of the dosing data respective to strength training programs.

Our analyses combined data either across exercise intervention types or across pain conditions likely contributing to variability in the data. It is possible that additional papers not included in Geneen et al. could impact the results of this study. Studies implementing large samples of specific pain populations testing a specific exercise intervention will need to be performed in order to accurately address the issue of dose in exercise therapy for chronic pain.

An additional limitation to our analysis was the potential for multi-collinearity. While the pain effect produced by the multi-collinear interaction terms may complicate the clinical application of prescribing exercise, the necessity to include these terms brings home the importance of considering all three dose measurements when prescribing an exercise regime. The initial multiple linear regression model which attempted to fit only the three main dose measurements resulted in a multiple linear regression equation in which only the DURATION coefficient was significant with R^2^ = 0.471. Removing the non-significant coefficients of FREQUENCY and TIME caused the R^2^ value to decrease substantially (R^2^ = 0.293), suggesting that TIME and FREQUENCY were somehow important when combined with DURATION. The resulting model brought their importance in focus by producing significant main effects and interaction effects, as well as strengthening the R^2^ value to 0.552.

### Clinical implications

Even in the context where exercise is generally beneficial, the lack of any explicit dosing knowledge may mean that the medical community is subjecting patients to unnecessarily high levels of exercise or missing the opportunity for additional therapeutic benefits. The lack of dosing data may also lead to reduced patient compliance if patients are prescribed a less-than-efficacious exercise therapy [162]. We hypothesize that, with special consideration for those persons with musculoskeletal pain, beginning exercise therapy at a lower dose and subsequently increasing physical activity as tolerated may be the most beneficial. Many chronic pain patients are not accustomed to exercising at a high level[163, 164] and may experience significant fear-avoidance behavior and kinesiophobia[165–167]. Others have recommended beginning exercise at a low intensity or dose and gradually progressing to a moderate intensity[168]. This “low and slow” approach to therapy may make patients more liable to adhere to exercise protocols.

Overall, multivariate linear regression modeling allowed us to evaluate the interaction of different aspects of exercise dose, as well as the impact of the significance of the original study on pain effect. Increasing exercise dose as defined as FREQUENCY per week, is predicted to be the most likely to cause significant pain relief for patients (**Table 4**). It is important to note that the predicted decrease in effect size due to increased frequency is countered by the positive interaction effect of the exercise duration by frequency. Because of this, the overall net effect is an increased analgesic effect. Some caution needs to be used in carrying this result to the clinic as it is based on modeling of existing data. Nonetheless, the model makes valuable and, more importantly, testable predictions that can be addressed in future randomized controlled trials. While the present study cannot identify optimal values for dosing an exercise program or for detecting a significant pain effect between treatment and control groups for chronic pain, these results do suggest that varying exercise dose as measured by TIME, FREQUENCY and DURATION significantly influences a study’s measured effect size. And while changing “frequency per week” results in the most influential analgesic effect, this dose measurement is naturally limited to multiples of 7 days per week. **Table 4** suggests that one might receive an analgesic effect by exercising a shorter amount of time daily; an exercise regimen that might be more acceptable to one experiencing chronic levels of pain and one that might be more amenable to sustained compliance. Furthermore, quality of life is an important outcome for pain patients. Even if exercise therapy is unable to effectively manage pain, it is essential to increasing health related quality of life in these patients by improving other aspects of their well-being including their physical fitness. Future studies should be mindful of incorporating these outcome measures when working to manage all aspects of the patient’s pain.

## Conclusions

Overall, this analysis of the existing literature demonstrated insufficient evidence for the presence of dose effects of exercise in relation to analgesia. Ultimately, the major problem in this area is that no studies identified in this analysis individually account for the dose of exercise in the trial. Specific randomized controlled studies with larger n’s, done in specific patient populations, and multiple doses are necessary to determine the effects of exercise dose on the efficacy of exercise for chronic pain conditions. Future studies should provide a high level of specificity in the prescribed dose of exercise by reporting the frequency of exercise, timespan of the session, intensity of the exercise and duration of the intervention. As a field, it is necessary to start incorporating multiple doses in exercise studies in order to obtain the best possible outcome for our patients. Based on our multi-variate analysis, idealized future studies should be performed testing varying frequencies (exercise bouts per week) of explicit aerobic exercise interventions that are performed less than 120 minutes per week for no more than 15 weeks to achieve optimal pain reducing effects. These studies should be performed in specific pain populations and should consist of specific exercise regimens due to the nature of the pathology of each chronic pain condition and the physiologic response to different forms of exercise.

## Supporting information

S1 File PRISMA checklist. Preferred reporting items for meta-analysis.
(DOC)
